# A Cyber-Physical Data Collection System Integrating Remote Sensing and Wireless Sensor Networks for Coffee Leaf Rust Diagnosis

**DOI:** 10.3390/s21165474

**Published:** 2021-08-13

**Authors:** David Velásquez, Alejandro Sánchez, Sebastián Sarmiento, Camilo Velásquez, Mauricio Toro, Edwin Montoya, Helmuth Trefftz, Mikel Maiza, Basilio Sierra

**Affiliations:** 1 RID on Information Technologies and Communications Research Group, Universidad EAFIT, Carrera 49 No. 7 Sur-50, Medellín 050022, Colombia; asanch41@eafit.edu.co (A.S.); ssarmien@eafit.edu.co (S.S.); cvelas31@eafit.edu.co (C.V.); mtorobe@eafit.edu.co (M.T.); emontoya@eafit.edu.co (E.M.); htrefftz@eafit.edu.co (H.T.); 2 Department of Data Intelligence for Energy and Industrial Processes, Vicomtech Foundation, Basque Research and Technology Alliance (BRTA), 20014 Donostia-San Sebastián, Spain; mmaiza@vicomtech.org; 3 Department of Computer Science and Artificial Intelligence, University of Basque Country, Manuel Lardizabal Ibilbidea, 1, 20018 Donostia-San Sebastián, Spain; b.sierra@ehu.eus

**Keywords:** coffee leaf rust, cyber-physical system, internet of things, mechatronic design, technological integration, remote sensing, wireless sensor networks

## Abstract

Coffee Leaf Rust (CLR) is a fungal epidemic disease that has been affecting coffee trees around the world since the 1980s. The early diagnosis of CLR would contribute strategically to minimize the impact on the crops and, therefore, protect the farmers’ profitability. In this research, a cyber-physical data-collection system was developed, by integrating Remote Sensing and Wireless Sensor Networks, to gather data, during the development of the CLR, on a test bench coffee-crop. The system is capable of automatically collecting, structuring, and locally and remotely storing reliable multi-type data from different field sensors, Red-Green-Blue (RGB) and multi-spectral cameras (RE and RGN). In addition, a data-visualization dashboard was implemented to monitor the data-collection routines in real-time. The operation of the data collection system allowed to create a three-month size dataset that can be used to train CLR diagnosis machine learning models. This result validates that the designed system can collect, store, and transfer reliable data of a test bench coffee-crop towards CLR diagnosis.

## 1. Introduction

Coffee, for over 1000 years and even today, has been one of the most consumed drinks around the world with more than 400 billion cups per year [1]. Among more than 100 existing coffee species, only two are used for the drink preparation, namely *Coffea arabica* and *Coffea robusta*. The first one, which is used to obtain a more aromatic and softer beverage, is best valued by the market and represents over 75% of the world production. The drink resulting from processing the second one, which is considered to have a stronger and more bitter flavor, represents the remaining 25% [2]. Moreover, each species subdivides into coffee varieties, each of them having characteristics that allow the creation of distinct aromas and tastes.

As a case study, we consider the case of Colombia, which is the third major coffee producer of the world [3]. Colombia is located on the Bean Belt, a strip across the globe where all coffee plants are grown [4]. The national production is concentrated on *Coffea arabica*, due to the mountainous topography of the country, which offers a suited combination of altitude, temperature, and rainfall for this species. Particularly, the most cultivated varieties of *Coffea arabica* in the country are Castillo, Colombia, Caturra, and Bourbon [5]. Depending on the selected variety and the post-harvesting process, the resulting product is offered in two different markets. One of them is the standard coffee market, which is guided by the international coffee price, and the other one is called the specialty coffee market, which has a premium above the standard price.

Regarding the phytosanitary problems on coffee crops, one of the main concerns is related to the presence of pests, such as the Coffee Borer Beetle, and diseases, such as the Coffee Brown Eye Spot and the Coffee Leaf Rust (CLR) [6]. For the diseases, the CLR is the most relevant one, in economic and pathological terms, at the national level. This disease presents a vertiginous expansion on the coffee plant and its surroundings, and it can cause massive defoliation on the whole crop [7]. As an example, in extreme cases, this disease has led to devastating losses in some Colombian regions reaching between 70% and 80% of the harvest [6].

It should be noted that the use of technology to support agriculture has made it possible to automate and optimize production. In this sense, sensors can be used both to monitor the machinery required for a plantation (e.g., performing predictive maintenance [8]) and to detect specific features of a crop and its ecosystem (e.g., non-invasive phenotyping in plant breeding [9]). Other applications of the use of sensors in agriculture may include precision irrigation, greenhouse instrumentation, and pest control [10].

It is noteworthy that, at the beginning of this research, the general objective was oriented to the early detection of the Coffee Brown Eye Spot disease through Remote Sensing (RS) with spectral reflectance data analysis. However, after carrying out the interviews with the Colombian coffee experts and producers, we realized that the mentioned disease was not as crucial or economically limiting as the CLR. The interviewees expressed that their main concern was the CLR and most of them even reported that they have been struggling with it over the last four years. Thus, and thanks to their recommendations, we decided not only to diagnose the CLR instead of the Coffee Brown Eye Spot disease, but also to integrate Wireless Sensor Networks (WSN). In that sense, the first step towards diagnosing the disease consisted of collecting reliable data regarding its development. Thereby, once the necessary data had been collected, it would be possible to create a diagnostic model based on such data. Therefore, this research presents the following two contributions: (i) The mechatronic design of a cyber-physical data collection system to collect and store data, integrating RS and WSN; (ii) a three-month dataset for CLR detection.

This paper is structured as follows: Section 2 explains some key concepts and describes related work by different authors, Section 3 presents the conceptual and detailed design of the data collection system, Section 4 shows the building and integration of the mechanical, electronic, and computing components and, finally, Section 5 states the conclusions, recommendations, and future work.

## 2. State of the Art

Different studies have been carried out involving technical methods and strategies for obtaining nutritional information of different types of crops [11], diagnosing diseases [12], and detecting pests [13]. Recently, an important concept has emerged called Precision Agriculture (PA). PA refers to an agricultural management concept that uses information and communications technology to observe, measure, and respond to specific crop variabilities. PA includes applying the correct treatment method at the right time according to the needs of the plants [14].

In PA, one of the current methods used to evaluate the features of different crops is called RS. RS relies on the interaction between materials and their electromagnetic radiation. It includes receiving radiation reflected from soil or plants to obtain valuable information, such as chlorophyll content, water stress, weed density, crop nutrients, and disease presence. These measurements can be made using airplanes, portable sensors, satellites, tractors, and drones [15].

Several authors [16,17,18] pointed out the importance of using high-quality portable devices to detect and control diseases in hard-to-reach sites. For example, Goel et al. [16] analyzed the detection of variations in the spectral response of corn (Zea mays) due to nitrogen application rate and weed control. For this reason, a hyperspectral sensor called Compact Airborne Spectrographic Imager is used to analyze the reflectance values of 72 bands in the range of 409 nm to 947 nm. These bands include visible light and external Near-Infrared (NIR) from the radiation spectrum. Their research demonstrated the potential of using hyperspectral sensors to detect weed infestation and nitrogen stress. Specifically, the most suitable wavelength bands for detection are found to be the wavelength regions around 498 nm and 671 nm, respectively.

In addition, a crop classification method employing the infrared and visible portions of the electromagnetic spectrum and low-cost cameras in a multi-rotor aircraft was proposed by Bolaños et al. [17]. This study is based on the identification of Normalized Difference Vegetation Index to assess health status and moisture content. Similarly, Chemura et al. [18] presented a method for predicting the presence of diseases and pest infestations early in coffee trees due to imperceptible water pressure. To this end, a handheld multi-spectral scanner with the visible and near-infrared regions is placed in an Unmanned Aerial Vehicle. Chemura et al. research is also related to irrigation planning based on the specific water needs of plants.

In addition to RS, based on smart farming techniques and the Internet of Things (IoT), which refers to the use of intelligently connected devices and systems leveraging data acquired by embedded sensors and actuators in machines and other physical objects [19], there is another popularly used method named WSN. WSN is responsible for real-time monitoring of different agricultural characteristics. It consists of multiple integrated, unattended devices called sensor nodes, which collect data at the site and wirelessly transmit it to a centralized processing station (called a base station). This station can store, process, and transmit data to the Internet, where a final user can analyze and transform it into relevant information and knowledge [20].

In this regard, Chaudary et al. highlighted the importance of WSN in the PA field by controlling and sensing the most relevant variables of a greenhouse using a microcontroller technology named Programmable System on Chip. This research examined the integration of wireless sensor nodes with high-bandwidth spectrum telecommunications technology, which proved helpful in determining the optimal irrigation strategy that meets crops’ specific needs. Moreover, the study recommended using reliable low-current consumption hardware for WSN applications because it improves farmers’ confidence to incorporate them into their crops [21]. Additionally, Piamonte et al. implemented a WSN prototype for monitoring an African Oil Palm disease called the Bud Rot. By using humidity, pH, light, and temperature sensors, their prototype measured climate change and soil factors to identify the presence of disease-causing fungi indirectly. This research concluded that the measurement results for the aforementioned non-biological factors had changed slightly, which, according to the researchers, indicates the possibility of detecting the Bud Rot [22].

The presented state of the art shows that RS and WSN are two widely used methods within PA due to their capacity to monitor different crop features and detect the presence of various anomalies.

## 3. System Design

Previous research was helpful to design a cyber-physical data collection system that could integrate both methods towards the CLR diagnosis. Applying the concepts and following the recommendations found in the state-of-the-art, it is possible to create a system capable of acquiring and remotely storing reliable data from diverse sources. The goal of such cyber-physical systems (CPS) is the characterization of a test bench coffee-crop regarding the changes induced by the disease at hand. The cyber-physical data collection system was designed following the Pahl and Beitz methodology [23]. The mechatronic design of the data collection system is presented in this section.

For the development of the system, requirements were elicited with the participation of Colombian Coffee Agricultures Association (CENICAFÉ) and University EAFIT. From EAFIT, the design team was composed by the Mechanical, Informatics and Electronic Engineers, as well as Biologists. The fulfillment of those requirements, which included, among others, building a test bench coffee-crop, emulating different agronomic conditions, and allowing the data acquisition, storage, and transfer, was the guideline for the design of the system. In that sense, this section describes, in a stepwise fashion, the use of the Pahl and Beitz methodology for achieving a data collection system that integrates RS and WSN towards the CLR diagnosis.

### 3.1. Main Requirements

First, all requirements were formalized, structured, and classified according to their characteristics and priority through the employment of the Product Design Specification [24]. The main requirement was measuring physicochemical features of the plants as well as capturing Red-Green-Blue (RGB) and multi-spectral images of the test bench coffee-crop for storing all this data locally and remotely. Other requirements were related to plants’ separation and irrigation, coffee variety to be used, construction materials, database type, and communication protocol with the field sensors.

### 3.2. Black Box Definition

**Figure 1:**
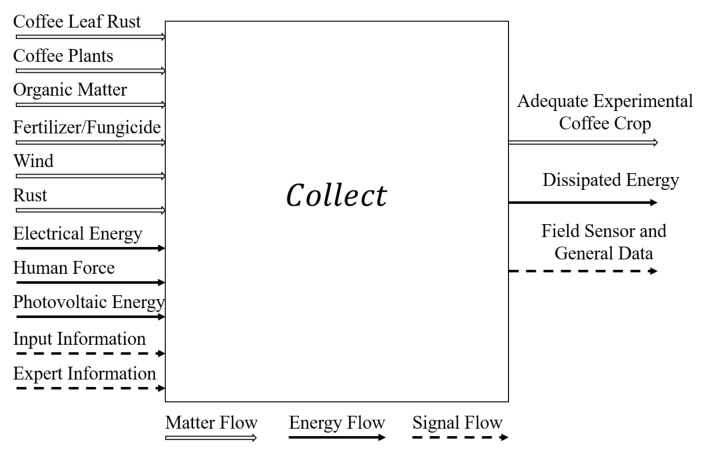
Black box representation of the cyber-physical data collection system.

The following step is to design a black box [25], which represents the primary function of the system to be developed. This primary function is to collect a set of inputs, transform them, and produce a set of outputs. As shown in Figure 1, the inputs and outputs are divided into three major flows: namely matter, energy, and signal. Regarding the inputs, the matter flow was composed by CLR, coffee plants, organic matter, fertilizer/fungicide, and wind; the energy flow was divided into electrical energy, human force, and photovoltaic energy; and the signal flow consisted of input information and expert information. At the output, the adequate experimental coffee crop dissipated energy as well as field sensors and general data records were obtained. These output data correspond to the main objective of this research, which is to create a system capable of collecting, storing, and transferring reliable data of a test bench coffee-crop towards the CLR diagnosis.

### 3.3. Functional Structure

After defining the Black box, the functional structure [26] was specified, breaking down the presented inputs and outputs and establishing, with a detailed understanding, the sub-functions required, and the pathway created by these. As a way of example, one of these sub-functions consisted of merging human force with the coffee plants to arrange the latter in the test bench coffee-crop, which impacts the posterior incorporation of the field sensors. The general pathway of the overall functional structure is described as follows.

The coffee plants were divided into four lots, where half of them were inoculated with *Hemileia vastatrix* [27], the fungus that causes CLR. For their agronomic management, fertilizer and fungicide were distributed and incorporated into all four lots. Then, each lot was isolated from the others to make them independent and, finally, the whole crop was integrated with the rain and wind emulation systems. Rainfall and wind speed were both monitored and regulated for the entire crop.

Furthermore, employing sensors in each lot, soil moisture/temperature, pH, illuminance, and environmental humidity/temperature were acquired. In addition, RGB and multi-spectral images were captured. To finish the data collection process, data were stored locally, pre-processed for cleaning purposes, and then sent to a remote server over the Internet. In addition, the collection process was monitored in real time through an IoT web platform.

### 3.4. Morphological Matrix and Candidate Concepts

Once the main function and the corresponding sub-functions were specified, the morphological matrix [28] was developed. Such a matrix illustrates different solution proposals for the implementation of each of the sub-functions exposed in the functional structure. The output of the morphological matrix consisted of two candidate concepts, Concept 1 and Concept 2, each made up of a different combination of the solution proposals. The concepts indicated two possible ways of building the data collection system, and they were elaborated with the purpose of evaluating them under different aspects and deciding which was the most appropriate for the objective at hand. The most relevant features for Concept 1 and Concept 2 included: (i) holes in tubes or sprinklers to emulate rain, (ii) a stepper motor or a servomotor to position the multi-spectral cameras over the lots, (iii) using normal pressure from the aqueduct or a pump to transport the water for irrigation, and (iv) a rotary arm or a single rail to capture images from multispectral cameras, respectively (see Table S1 from supplementary material uploaded at MDPI platform and at provided link in Supplementary Materials section). The resulting candidate concepts were then evaluated by using a scoring system, which calculates a weighted average of a set of pre-selected evaluation criteria. These weights were established according to the previously defined PDS and the design team expertise. As a result, the final concept is selected. As shown in Table 1, Concept 1 resulted as the selected concept, with an approval of 78% against 74% of Concept 2. The cyber-physical data collection system was built based on the winning concept. CPS are a new class of engineered systems which offer close interaction between cyber and physical components [29]. It should also be noted that the chosen concept was slightly modified following some improvements proposed by CENICAFÉ and the design team to better fulfill the initial requirements.

### 3.5. Final Concept

**Figure 2:**
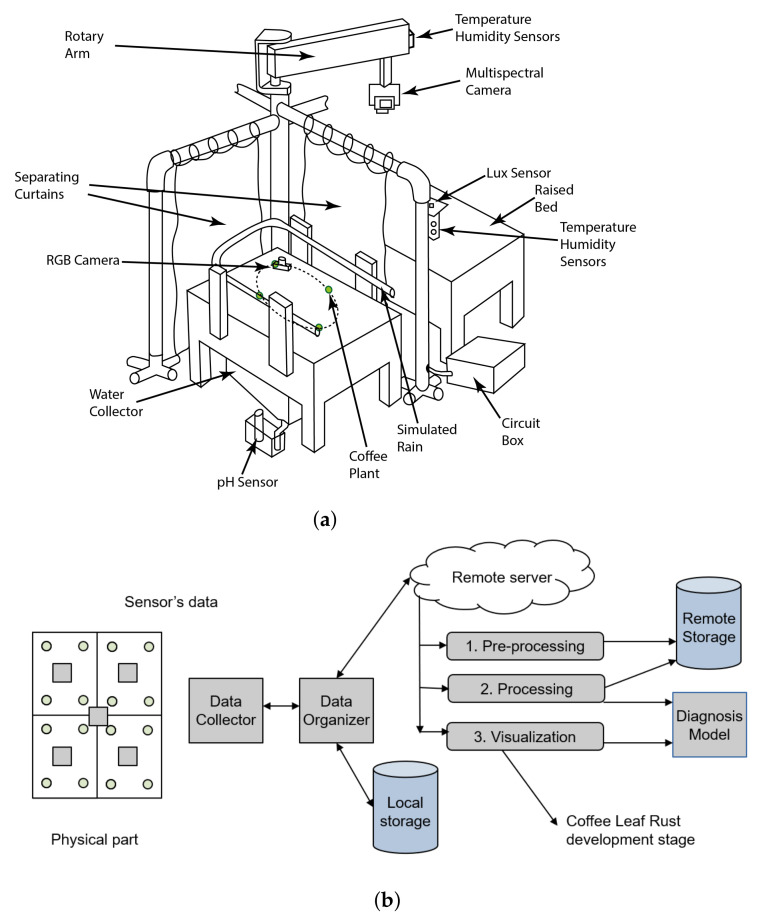
Final concept sketch for the data collection system: (**a**) of the physical part; (**b**) of the cybernetic part.

Figure 2a shows a sketch of the final concept for building the physical part of the system. This concept is composed of four raised wooden beds representing the lots and separated by four plastic curtains, a rotary arm holding the multi-spectral cameras, a rain system which irrigates the lots, and a circuit box with the necessary elements to interact with the electronic components. Figure 2b shows a sketch of the cybernetics part of the design, which includes a data collector for joining the data coming from the test bench coffee-crop and a data organizer, which structures and saves it on the local storage for its posterior transfer to a remote server located at EAFIT University.

### 3.6. Mechanical Design

The mechanical design considered four identical crop lots, each one housing four coffee plants under different development stages of the disease. Each lot had specific structures designated to place the sensors for pH, illuminance, soil moisture, soil temperature, environmental humidity, and environmental temperature. In addition to the sensors, a rotary platform, holding a rack-pinion mechanism and containing a slider extension, three micro-switches, a mini-DC motor, and a digital servo, was also designed for each lot aiming at driving an RGB camera close to each plant for capturing images from the bottom of the leaves. In addition, each lot had a filtering point, which directed the residual water into a container where the pH-meter was placed.

Furthermore, a rotary arm was designed to place two multi-spectral cameras and to be able to move them above the four crop lots for capturing images from the upper side of the leaves. One of the cameras had an RGN filter (Red-850 nm, Green-660 nm, Near Infrared-550 nm), whereas the other one had a RE filter (Red Edge-735 nm). Both filters were suitable for assessing the presence of plagues and diseases (in particular the CLR) in crops [30,31]. Moreover, since the coffee plants needed a suitable environment to grow, a rain system was also designed for irrigation purposes. This system was controlled through an open/close command that could change the state of a corresponding solenoid valve according to a pre-defined rain schedule.

### 3.7. Electronic Design

To collect data from each crop lot using field sensors, an electronic design of the system was required. Sensors, actuators, interfaces, power supplies, two (2) Arduino Mega microcontrollers, one (1) Raspberry Pi microcomputer, and an electrical cabinet composed this electronic design. These electronic components were connected and integrated to support the cybernetic part of the data collection system. In what follows, each component is described.

#### 3.7.1. Arduino Mega Microcontrollers

One of the Arduino Mega microcontrollers (named Arduino 1) collected lot data. Thus, four pH-meters, four illuminance sensors, four soil moisture sensors, four soil temperature sensors, and one environmental humidity/temperature sensor were connected to it. In addition, the Arduino controlled the movements of the central rotary platform. For its part, the other Arduino Mega (named Arduino 2) was considered for the general data collection, having the tasks of activating/deactivating the rain system, communicating with the flow and wind sensors, as well as moving the rotatory arm over the lots. Both Arduino Mega microcontrollers were communicated with the Raspberry Pi via USB.

#### 3.7.2. Raspberry Pi

The Raspberry Pi was responsible for orchestrating the sequence of steps during each data collection routine, storing the gathered data locally, and transferring it to a remote server over the Internet. For that purpose, in addition to being communicated with both Arduino Mega microcontrollers, four RGB and two multi-spectral cameras were connected to it via USB. Thereby, the Raspberry Pi was able to trigger the different electronic devices and obtain the results. Finally, to send data to the remote server, an outdoor 4G LTE router was used to facilitate remote connectivity from the Data Collection System location.

#### 3.7.3. Electrical Cabinet

The design of an electrical cabinet was required for the distribution and organization of all electronic components. This cabinet had an IP5 minimum environmental protection due to the system exposure to the greenhouse’s harsh conditions. Furthermore, a current security breaker was also included to protect the components from a peak current over 10 A, considering that the total consumption of the data collection system was about 7 A. Additionally, protection fuses were proposed for each power supply and actuator to mitigate damages, and one 9 V/1 A AC/DC adapter was considered for each microcontroller to avoid problems related to low current values.

The Raspberry Pi and the Arduino Mega microcontrollers were connected through a Master–Worker network architecture. The connections were implemented by a serial interface.

### 3.8. Software Design

The software design was essential to detail how the physical components communicated with the cybernetic part of the system to collect and transfer the data properly. In that sense, it is essential to explain the principal functions, commands, components, architectures, content specifications and platforms, which were thought as necessary for managing the incoming and outgoing data flow.

#### 3.8.1. Data Acquisition, Conditioning, and Storage Routines

Several functions regarding the acquisition, conditioning, and storage of the readings of the electronic devices connected to Arduino 1 and Arduino 2 were defined. Arduino 1 functions were in charge of collecting, conditioning, and storing the data proceeding from the pH, soil temperature/moisture, illuminance and environmental temperature/humidity sensors, and controlling the servomotor angle and arm’s extension to position the RGB camera of each lot. Arduino 2 functions included collecting, conditioning, and storing readings from the flow rate and wind speed sensors and controlling the direction and destination of the global rotary arm that holds the multispectral cameras. The programs of both Arduino Mega microcontrollers were designed to respond to specific commands sent by the Raspberry Pi.

#### 3.8.2. Main Orchestration Program

A main, global program run by a Raspberry Pi in charge of orchestrating every step of the data collection routines and automatically executing such a process seven times a day was defined. Such an orchestration program was also in charge of activating/deactivating the rain system according to a pre-set schedule. This pre-set schedule included the raining days of year 2018. In addition, the program implemented by the Raspberry Pi was in charge of receiving and organizing the collected data from the Arduino Mega microcontrollers, triggering the RGB and multi-spectral cameras, storing everything locally in a structured way and transferring it to a remote server, named Academic Data Center (ADC), over the Internet via Secure File Transfer Protocol (SFTP). Finally, this program reported the current state to Thingworx (IoT platform, see subsection (iii)) during each routine. The software technologies selected for the implementation of this program were Python 3.5.3, OpenCV 3.4.1 [32], RPi.GPIO 0.6.3 [33] and MongoDB [34].

#### 3.8.3. Thingworx

*Thingworx* is a complete software platform designed for the Industrial IoT [35]. It was used to develop a dashboard, to remotely monitor the field and general data in real-time. This software platform was installed in the ADC. The ADC is the remote server used for remotely storing the collected data from the CPS to replicate the Raspberry Pi’s local storage. This server is hosted by the Computer-Science Department of *EAFIT University* and is composed of 72 Cores, 512 GB RAM, 4 TB Storage, 2 GPU Tesla K80, and an Ubuntu 18.04 Operating System. It can be accessed over the Internet through a *Virtual Private Network (VPN)* connection.

**Figure 3:**
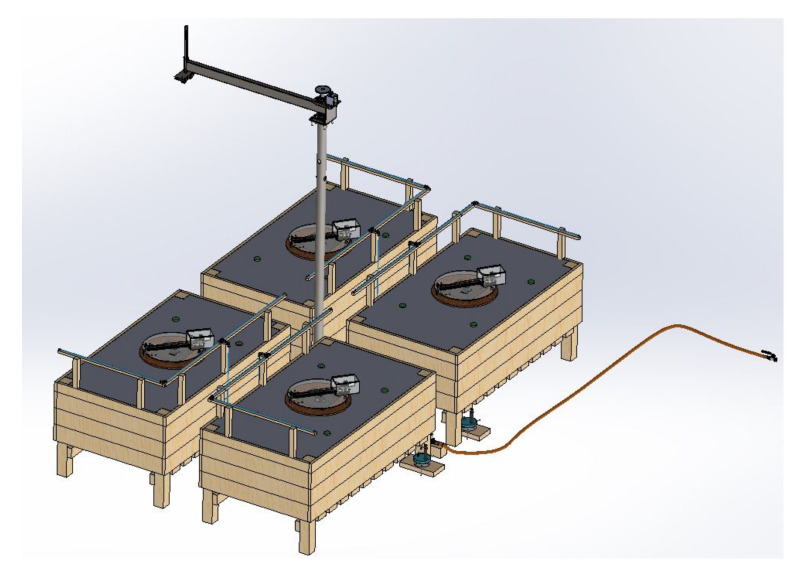
Final 3D CAD of the data collection system’s physical part.

The use of the Pahl and Beitz methodology allowed for evolving from the elicitation of the initial requirements towards the achievement of the final and detailed design of a data collection system that integrates RS and WSN. The final 3D Computer-aided design (CAD) of the data collection system’s physical part is shown in Figure 3.

**Figure 4:**
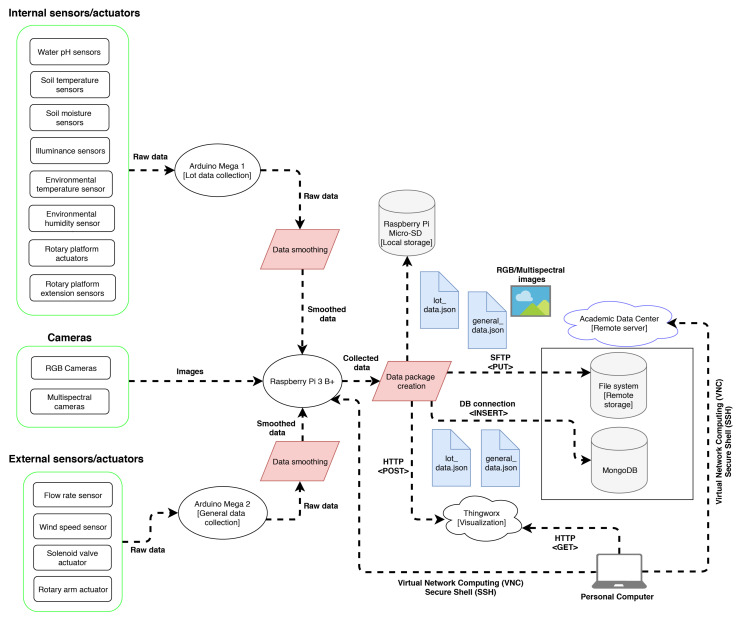
Final design of the data collection system’s cybernetic part.

Figure 4 shows the final design of the data collection system’s cybernetic part, which explains the pipeline for the execution of the data collection system.

## 4. Results

The application of the Pahl and Beitz design methodology results in the target system solution. Precisely, the result of this research work corresponds to the solution obtained by applying the Pahl and Beitz methodology, i.e., the target system, i.e., a cyber-physical data acquisition system capable of obtaining a dataset suitable for use in the early detection of CLR. Therefore, the following describes the results obtained, i.e., the system built, which was first represented by a 3D-CAD model.

### 4.1. Mechanical Components

These included the installation of a set of curtains, which were planned to work as a separation between the four crop lots. In addition, the assembly of the rotary arm structure was carried out. Finally, a separate structure for holding the multi-spectral cameras container was fixed.

Additionally, due to the emulated rain conditions that the prototype would be subjected to, immunized wood was chosen for the construction of the coffee crop lots, since it is resistant to moisture. At the bottom of each lot, a mesh in conjunction with a plastic tarp was installed to contain the soil. Furthermore, a slope was built within each lot using soil and impermeable plastic with the purpose of driving the residual water into the pH measurement system.

Regarding the rain system, all accessories related to the main pipeline for the incoming water source were installed over immunized wooden blocks. In addition, the whole wooden base was buried to keep the structure fixed. In addition, five supports were also installed on two adjacent sides of each lot for mounting a wooden L-structure over them. That structure served as a base for three additional hoses with small perforations, which would distribute the water within the lot producing the rain effect.

Succeeding the plants’ irrigation, the slope, which was built within each lot using soil and impermeable plastic, was helpful to drive the residual water into the filtering point located in one corner of each coffee crop lot. There, a hose was connected to lead the water to the container for the pH measurement.

Concerning the assembly of the rotary platform, several acrylic pieces were cut, including the rotary platform, rack, pinion, support for the rack, base, and protection for the RGB camera, supports for the mini-DC motor and digital servo and mechanical end stop for the rotary platform’s extension. To achieve the movement of the latter, a drawer slide was installed below this extension, and, therefore, the camera displacement towards the coffee plants could be achieved. Furthermore, before placing the rotary platforms in the center of each lot, steel plates were assembled to the supports of the digital servos and four levelers were screwed to the corners of each plate to put them underground for fixing the whole structure. Lastly, each platform was placed on top of a wooden base to mitigate the terrain’s instability.

### 4.2. Electronic Components and Their Integration

Following the construction of the mechanical components, the electronic integration was executed to complete the system’s physical part. For that purpose, each sensor and actuator were tested, calibrated, and connected to the corresponding micro-controller. In addition, individual connectors with a thick silicon protective layer were added to each of them to keep their metal terminals safe from the harsh conditions, which would include high temperatures, soil, and water from the rain system on a regular basis.

Similarly, the sensors’ calibration process was carried out to ensure the correct measurement and reliability of the data to be collected. Some sensors were already pre-calibrated at the factory (e.g., ambient temperature and humidity sensors) while others required calibration. For example, one of these sensors was the pH sensor, for which two buffer solutions with exact pH values were used. The sensor was adjusted to the same measured pH value as the buffer solution using the included potentiometers in the sensor’s interface. The calibration of the RGB cameras was carried out by manually adjusting the lens’s focus with respect to the leaves of the coffee plant to obtain a correct image sharpness.

After verifying the proper functioning and calibration of the sensors and actuators in the laboratory, along with the micro-controllers, they were merged into the corresponding mechanical structures to form the integrated components, which were required for the general operation of the cyber-physical data collection system. In addition, to protect, contain, and distribute the completely intermediate electronic devices (Raspberry Pi, Arduino Mega microcontrollers, USB-hubs, power supplies, and interfaces), an electrical hermetic cabinet was employed. To this end, the cabinet was subdivided into different sections, and it was also tailored for offering protection against dust, water, and a possible peak current. After installing it, the sensors, actuators, and cameras were connected to their corresponding place inside the electrical cabinet, guiding their cables through impermeable PVC pipes coming from the lots.

### 4.3. Software Components

Once all electronic components were duly tested and the respective drawbacks were successfully solved, it was possible to send commands to the Arduino Mega microcontrollers and trigger the cameras from the Raspberry Pi with the objective of verifying the communication, checking that the desired actions were executed and validating the results. Thereby, the integration of the mechanical and electronic components, along with the ability to control the system from the Raspberry Pi, was successfully proven.

**Figure 5:**
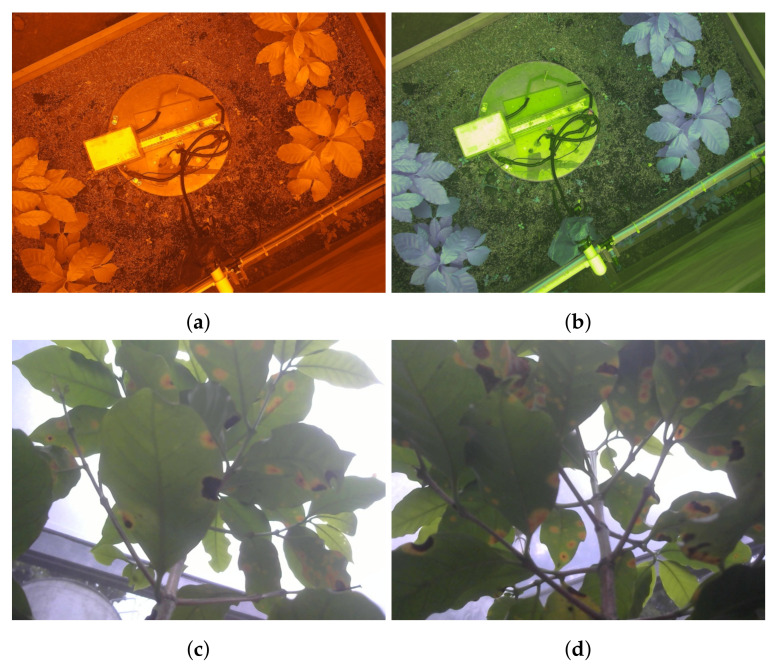
Example of generated files after data collection routine: (**a**) RGN image from lot 1; (**b**) RE image from lot 1; (**c**) RGB image from plant 3, lot 3; (**d**) RGB image from plant 4, lot 3.

For its part, the cybernetic part of the data collection system began with the implementation of the communication between Raspberry Pi and Arduino microcontrollers. It was achieved through the development of an Arduino communicator component, which established two separate serial connections and grouped the responses of the micro-controllers into single programming objects that could be subsequently structured and stored. Moreover, a data sub-directory creator component was developed for creating a sub-directory within the main data directory using the timestamp at which each data collection routine began. This component was also in charge of generating the proper internal structure for the files, which consisted of one sub-directory for each lot and another one for the general data. With respect to the files, different software components were also implemented to capture the RGB and multi-spectral images of the plants and write corresponding JSON files with the collected data and the paths to those images. Figure 5 shows an example of some of the generated files after concluding a routine.

In addition, a data visualization dashboard was developed using Thingworx according to the presented design for monitoring the current state during each routine. Thingworx’s appearance was preserved, and it was accessible through a Uniform Resource Locator (URL) with user-password authentication. Every used widget for creating the dashboard had a unique identifier so that it was possible to target each of them separately for updating their values.

After a routine finished, another software component, named data uploader, was responsible for transferring the collected data to the ADC over the Internet using a Wi-Fi connection. Consequently, the procedure to verify that the result was satisfactory consisted of checking whether the files uploaded to the ADC were identical to those stored locally in the Raspberry Pi.

### 4.4. Global Integrated System

Finally, the integration of the mechanical, electronic, and software parts led to the construction of a complete functional cyber-physical data collection system. The approximate total cost of the system implemented with all its components was 4863 USD. The total for the electronic and computer components were 3535 USD and for the mechanics 1328 USD. The most expensive components, in general, were the multispectral cameras with a cost of 1318 USD. It is estimated that, by implementing this system, followed by integrating an ML-based early CLR detection predictive model, the current harvesting losses due to this disease (e.g., 70% to 80% in Colombia) could be halved. Regarding maintenance costs, it is similarly estimated that these will be below 1% of net gains, which, considering estimated savings, is entirely affordable. Finally, the system’s lifetime is estimated to be five to ten years, which is usual in this kind of equipment and is within the standards of amortization periods. Nevertheless, these figures should be validated for a full-scale implementation of the system.

After completing the integration and construction of the data acquisition system mentioned above, a final test and calibration of each system component’s operation was performed, which is essential to ensure the system’s operation’s reliability. For example, one part of this process included the precise adjustment of the robotic arm position with respect to each plants’ lot for taking the multispectral photos, where each position was stored in the program to perform the data collection routine. Having performed the final system’s calibration, a data collection routine was executed for three months. The Data Collection System recorded crop’s cameras and sensors information from each lot seven times per day at different moments (with and without sunlight). It must be noted that, although the data storage occurred seven times per day, the system was acquiring and monitoring (Thingworx) the data in real-time, with a sampling period of 3 s. In addition to the data collected by the system, a biologist team evaluated and labeled daily in a separate file the current development stage of the CLR of each data collection system lot. The output of this routine generated a dataset comprising 603 RGN files (~153 MB), 641 RE files (~177 MB), 730 RGB files (~196 MB), and 672 sensor data (JSON) files (~1.12 MB), which were ready to be used for diagnosing the CLR development stage by training a Machine Learning model.

The operation of the current data collection system allowed the creation of a three-month size dataset. This dataset was used to train a deep learning model based on an ensemble algorithm integrating three convolutional neural networks and a multi-layer perceptron fed by RGB, RGN, and RE images; and Wireless Sensor Network data, correspondingly. This model was used to classify the early stage of CLR of a coffee crop (from 0 to 4), obtaining an F1-score of 0.775 [36].

## 5. Conclusions

This paper presents the mechatronic design of a cyber-physical data collection system, which integrates RS and WSN on a test bench coffee-crop. It is capable of automatically collecting, structuring, and locally and remotely storing reliable multi-type data from different field sensors (pH, soil moisture/temperature, illuminance, and environmental humidity/temperature), RGB and multi-spectral cameras. In addition, a data visualization dashboard was implemented to monitor the data collection routines in real time. This result represents a first step towards the CLR diagnosis on the *Caturra* variety.

The correct operation of the data collection system allowed for creating a three-month size dataset, which contains sensors and camera data required for creating a CLR development stage model. This result validates that the designed system can collect, store, and transfer reliable data of a test bench coffee-crop towards the CLR diagnosis.

For future work, this data collection system may be useful for measuring and recording different characteristics from other types of crops. In addition, and regarding the CLR, the data acquired through this system can be exploited for analyzing how the crop responds (in physicochemical and visual terms) according to the presence of the disease. It could be considered, for instance, to implement Artificial Intelligence techniques, such as Computer Vision and Deep Learning, to create a model based on the collected data for effectively diagnosing the CLR.

The current development is intended to be used as a test laboratory for plant experiments, which means that the obtained results are limited to a sample of a real crop plantation. As future work, a scalability, cost, and power consumption analysis could be carried out to turn the test laboratory into a full-scale mobile system. No relevant limitations are identified; however, employing drones and land robots are considered a technological requirement. Regarding drones, multispectral cameras (RGN and RE), which show the CLR in a distinct color from a top view of the crop, should be used. Concerning land robots, to effectively detect the CLR, they should be equipped with RGB cameras to monitor CLR’s yellow spots under the coffee leaves and land sensors (e.g., pH, temperature, humidity, soil moisture, and luminance). This research work will be helpful to size the optimal number and type of sensors required by such a full-scale implementation.

## Figures and Tables

**Table 1:** Concept Scoring. ${}^{a}$ Value scale (score between 0–4); 0 = Not satisfied, 1 = Acceptable, 2 = Sufficient, 3 = Good, 4 = Totally satisfied.

N${}^{\circ }$	Evaluation Criteria	Relevance (%)	Solutions ${}^{a}$	
			Concept 1	Concept 2
1	Functionality	11	4	3
2	Simplicity	5	3	4
3	Fulfilment of requirements	10	3	2
4	Robustness	3	4	3
5	Fabrication	7	3	3
6	Assembly	6	3	2
7	Reliability	9	3	3
8	Low cost	7	3	3
9	Expert criteria	6	3	3
10	Crop management	7	3	3
11	Maintainability	3	2	3
12	Performance	8	2	3
13	Usability	5	3	3
14	Testability	3	3	2
15	Availability	10	4	4
	Weighted average		3.13	2.96
	Total score	100	78%	74%

## Data Availability

This paper has a three-month dataset generated by the CPS Data Collection System, available at https://ieee-dataport.org/documents/coffee-leaf-rust-dataset (accessed on 8 July 2021).

## Supplementary Materials

The following is available online at https://www.mdpi.com/article/10.3390/s21165474/s1, Table S1: Morphological Matrix.

## Abbreviations

The following abbreviations are used in this manuscript:

ADC	Academic Data Center
CLR	Coffee Leaf Rust
CPS	Cyber-Physical Systems
NIR	Near Infrared
IoT	Internet of Things
PA	Precision Agriculture
RE	Red Edge
RGB	Red Green Blue
RS	Remote Sensing
SFTP	Secure File Transfer Protocol
URL	Uniform Resource Locator
VPN	Virtual Private Network
WSN	Wireless Sensor Networks
