# Supplementary material for: A Cyber-Physical Data Collection System Integrating Remote Sensing and Wireless Sensor Networks for Coffee Leaf Rust Diagnosis
Source: Sensors (Basel). 2021 Aug 13;21(16):5474. doi: 10.3390/s21165474 (PMC8401721; doi:10.3390/s21165474)
Supplement: Supplementary file 1 [file sensors-21-05474-s001.zip › sensors-1318148-supplementary.pdf]

Table S1. Morphological Matrix.

→ CONCEPT 1 → CONCEPT 2

	FUNCTION CARRIER			
**Function**	**PROPOSAL 1**	**PROPOSAL 2**	**PROPOSAL 3**	**PROPOSAL 4**
1. Group coffee plants	"Almacigo" group 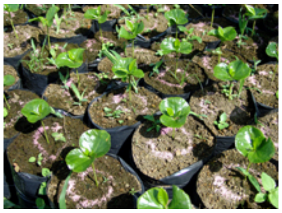	Coffee group with diverse ages 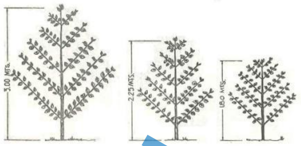	Coffee group in production stage 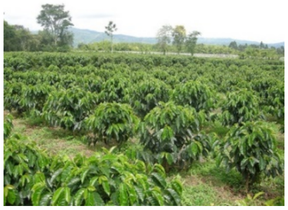	Coffee plants between "almacigo" and production stage 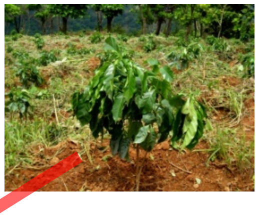
2. Store organic matter	Plant pots 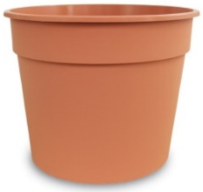	Plant bags 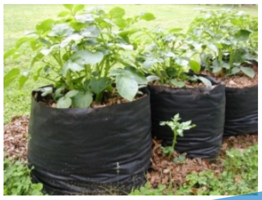	Crop beds 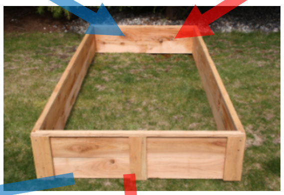	Regular crop disposal 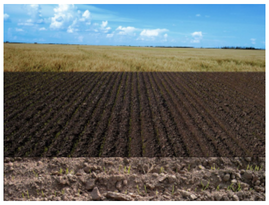
3. Store fertilizer and fungicide	Bottles 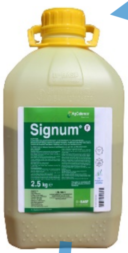	Tanks 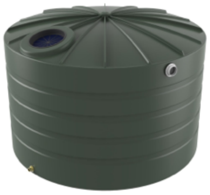	Spray 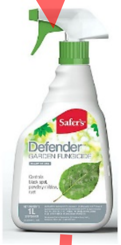	
4. Transport H_2O	Pumping 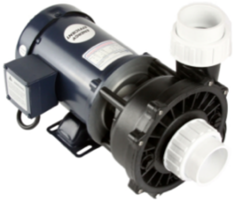	Gravity from tank 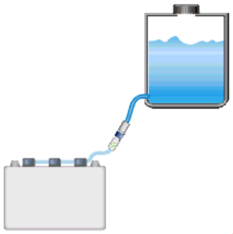	Normal pressure from aqueduct system 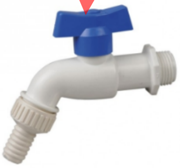	
5. Transform electrical energy	DC motor 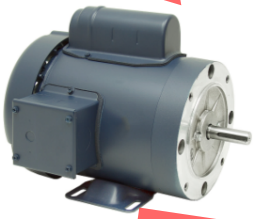	Stepper motor 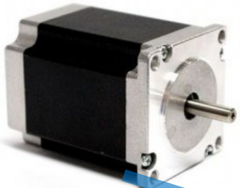	Servomotor 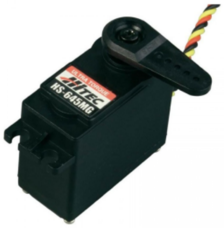	
6. Combine coffee plants with organic matter	Regular crop 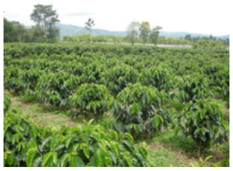	Single plant care 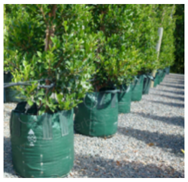	Raised garden beds 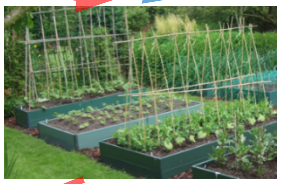	

7. Distribute fertilizer and fungicide	Spray 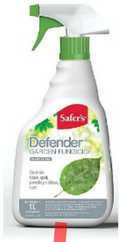	Pressurized knapsack 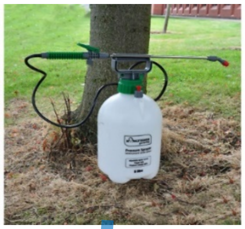		
8. Simulate rain with H_2O	Holes in tubes 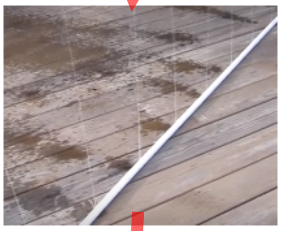	Sprinkler bottom-up 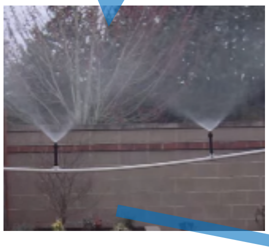	Sprinkler top-down 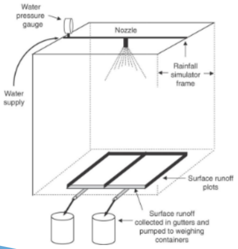	
9. Divide coffee plants with organic matter	Wood, class IV, penetration index = 9 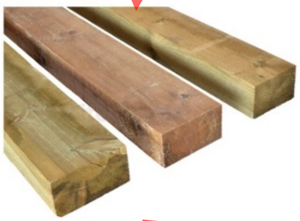	Acrylic 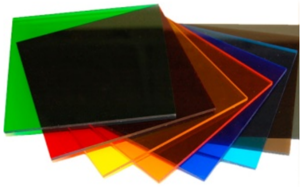	Bricks and cement 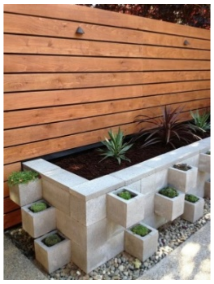	Plastic, polyethylene and polipropilene. 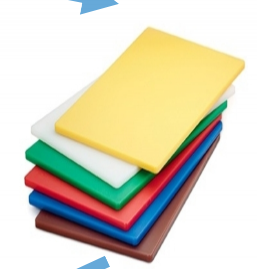
10. Inoculate Coffee Leaf Rust	In-vitro 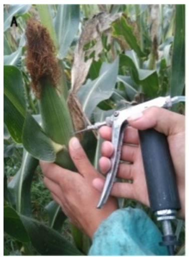	Natural (wind) 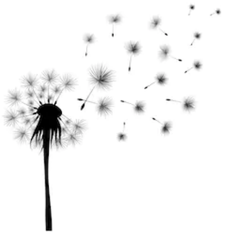	Rub with Coffee Leaf Rust 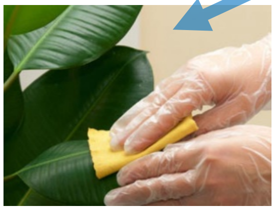	Brush 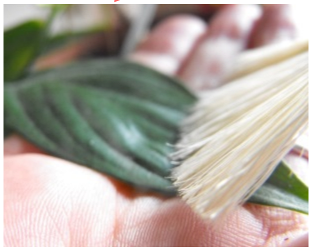
11. Assemble inoculated and non-inoculated coffee plants	One single piece divided 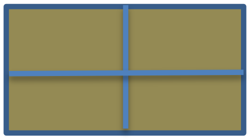	Independent lots 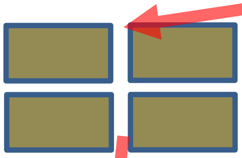	Pairs of lots 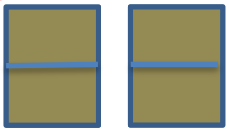	
12. Incorporate fungicide and fertilizer with coffee plants	Sprinkler 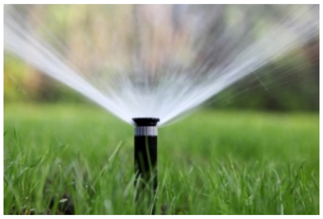	Spray 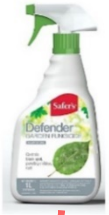	Pressurized knapsack 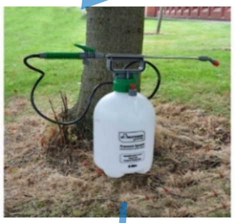	Holes in tubes 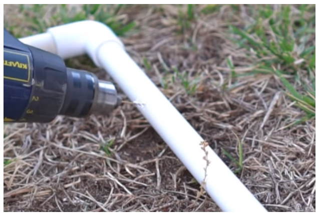

13. Perceive wind velocity	Ultrasonic wind sensor 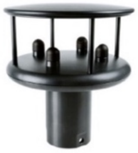	Dual wind sensor 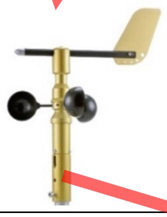	GPRS weather station 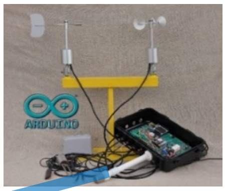	
14. Perceive volumetric flow rate	Flow meter 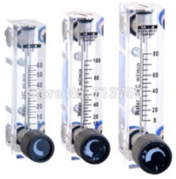	Pluviometer 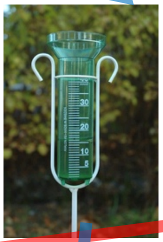	Electromagnetic flow meter 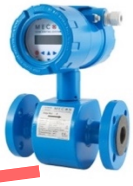	
15. Position multispectral camera	Stepper motor 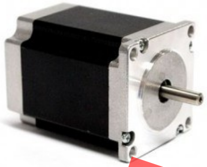	Servomotor 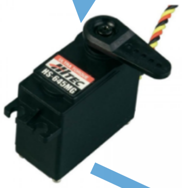	Manual 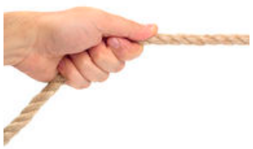	
16. Position RGB camera	Movement by chains 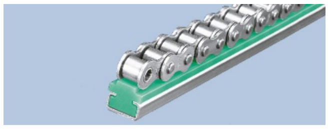	Curved rails 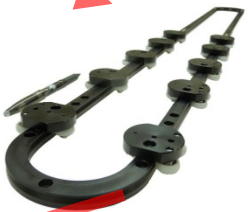	Endless screw 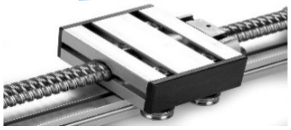	Small car positioning 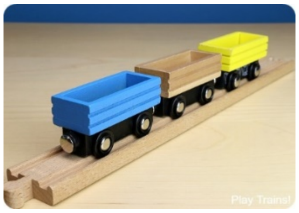
17. Isolate each lot of coffee plants	Plastic curtains 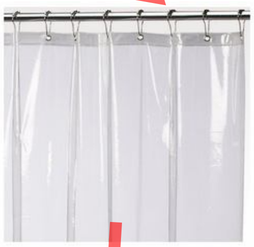	Fabric curtains 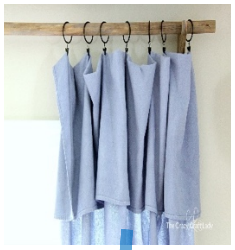	Blinds 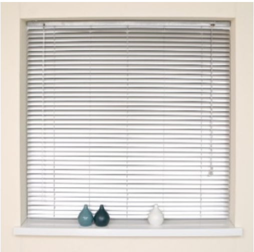	
18. Combine simulated rain with isolated lots of coffee plants	Holes in tubes raised from the lots 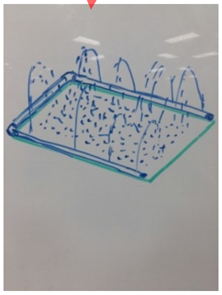	Sprinkler above the lots 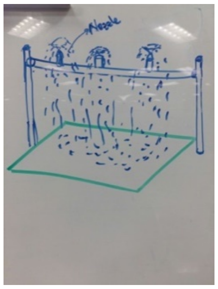		

19. Integrate expert info with isolated lots of coffee plants	Remote monitoring 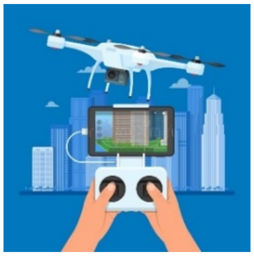	Visual inspection 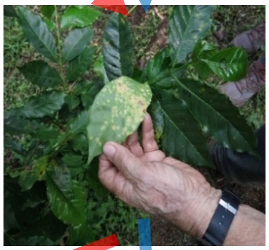		
20. Perceive pH in isolated lots of coffee plants	Type drainage 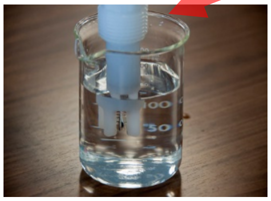	Type soil 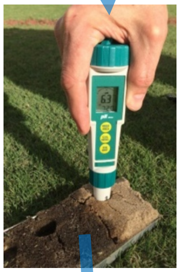	Type general 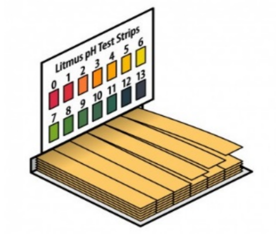	
21. Perceive lux in isolated lots of coffee plants	Lux sensor 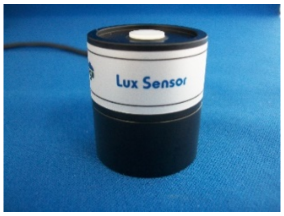	Digital luminosity sensor 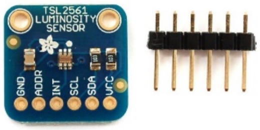	LDR 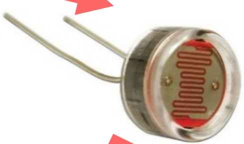	
22. Perceive humidity in isolated lots of coffee plants	Soil moisture capacitive sensor 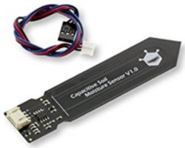	DHT-33 (x2) 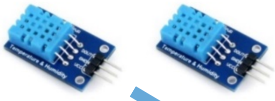	SHT-10 humidity sensor 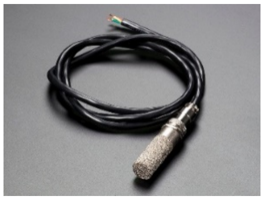	5TE / DHT-33 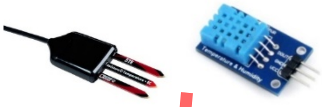
23. Perceive temperature in isolated lots of coffee plants	Type soil 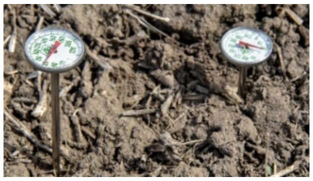	Thermometer 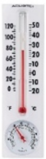	DHT-33 (x2) 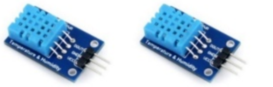	5TE / DHT-33 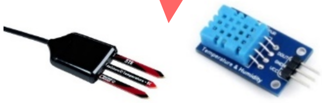
24. Perceive electrical conductivity in isolated lots of coffee plants	Conductivity electrode 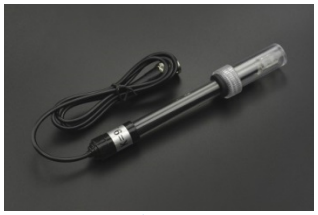	5TE 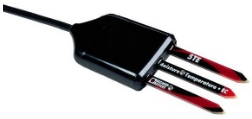		

25. Capture images from multispectral camera	Single rail 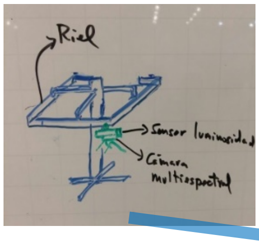	Rotary arm 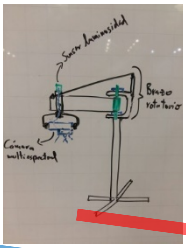	Manual 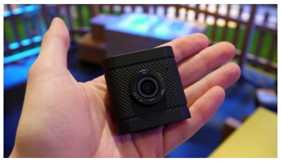	
26. Capture images from RGB camera	Zig-zag rail 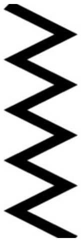	Straight rail 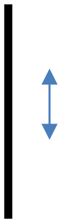	2-axis rail 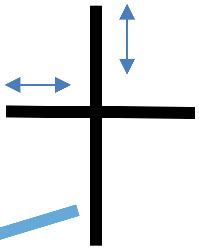	Elliptical rail 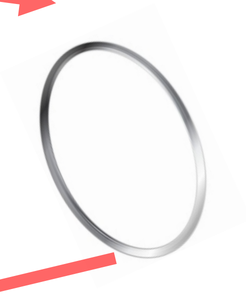
27. Cluster sensors' readings in a package	One package for each lot 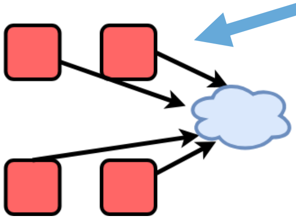	One package for the entire crop with lot ID 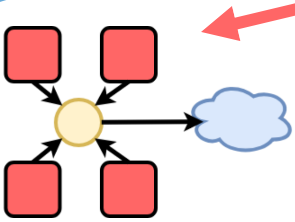		
28. Store collected data locally	USB 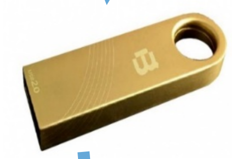	Micro-SD 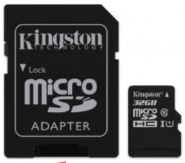		
29. Send data package to remote server	WiFi 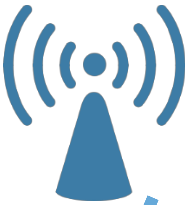	XBee 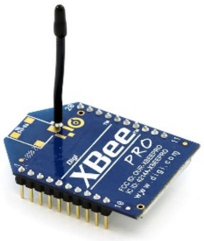	Bluetooth 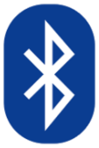	
30. Pre-process data package	Missing values to zeros 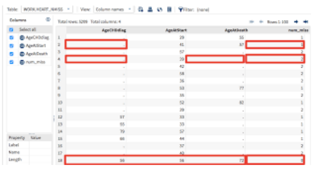	Normalization 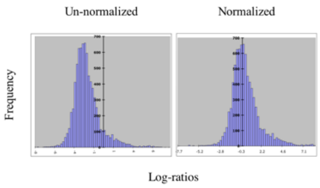	Remove records having missing values 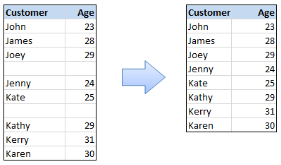	

31. Store pre-processed data package	Remote database 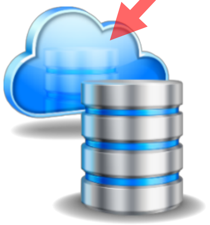	Local database on the remote server 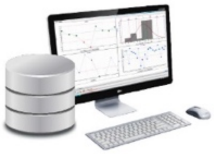		
32. Divide data into training, validation and test sets	Random division 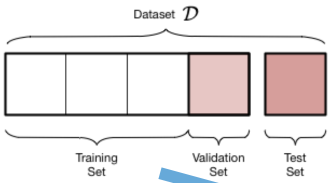	Balance across classes + Shuffle 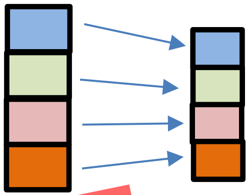		
33. Process training dataset	Deep Neural Network 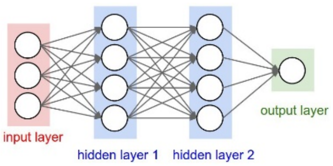	Support Vector Machines 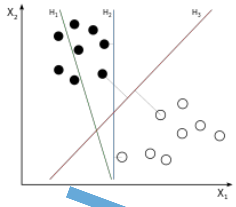	Random forest 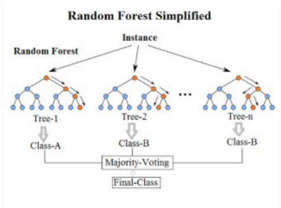	Clustering 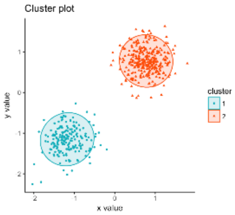
34. Evaluate the candidate model with the validation dataset	Hinge-loss 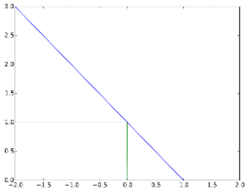	Cross-entropy 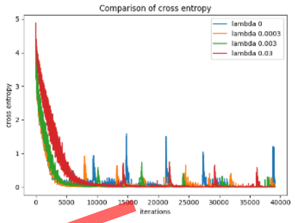	Mean-Squared-Error $\mathrm{MSE} = \frac{1}{n}\sum_{i=1}^{n}(y_i - \tilde{y}_i)^2$	
35. Visualize the results through a simple interface using some test data	Develop a web interface 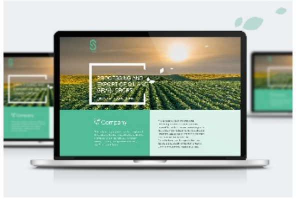	Develop a software to automatically send e-mails 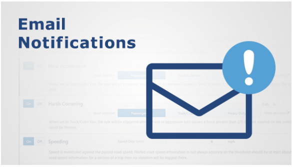	Develop an app 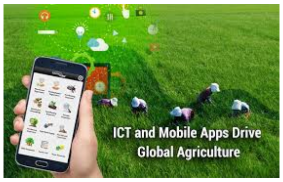	Use Thingworx PaaS<br>thingworx

References

1 – Proposal 1: “Cultivemos café / Almácigo,” Quienes Somos, 15-Dec-2015. [Online]. Available: https://www.cenicafe.org/es/index.php/cultivemos_cafe/almacigo/Almacigo. [Accessed: 03-Feb-2019].
1 – Proposal 2: Cultivo de platano - Anacafé. [Online]. Available: https://www.anacafe.org/glifos/index.php/Variedades_de_cafe. [Accessed: 03-Feb-2019].
1 – Proposal 3: “Coffee Plants of the World,” Specialty Coffee Association. [Online]. Available: https://sca.coffee/research/coffee-plants-of-the-world/. [Accessed: 03-Feb-2019].
1 – Proposal 4: Ray, “Vacation Planning,” Costa Rica Guide. [Online]. Available: https://costa-rica-guide.com/photos/coffee-growing-picking/. [Accessed: 03-Feb-2019].
2 – Proposal 1: Homepage. [Online]. Available: https://www.gsdistribution.ca/hc-canada. [Accessed: 03-Feb-2019].
2 – Proposal 2: J. Poindexter, “Growing Potatoes: A Beginner's Guide to Planting Big & Healthy Potatoes,” MorningChores, 02-Nov-2018. [Online]. Available: https://morningchores.com/growing-potatoes/. [Accessed: 03-Feb-2019].
2 – Proposal 3: “Raised Garden Beds,” USA Garden Company. [Online]. Available: http://www.usagardencompany.com/raised-garden-beds.html. [Accessed: 03-Feb-2019].
2 – Proposal 4: “Pics for you evety day,” King Yu of Xia Dynasty also known as Xia Yu Wikipedia t. [Online]. Available: http://naturtreenspicerx.pw/Healthy-soil-healthy-plants-healthy-people-articles-Soil-carbon.html. [Accessed: 03-Feb-2019].
3 – Proposal 1: “Signum Fungicide 2.5kg,” ProGreen. [Online]. Available: https://www.progreen.co.uk/signum-fungicide-2-5kg. [Accessed: 03-Feb-2019].
3 – Proposal 2: Prabha Polycolor Pvt Ltd. [Online]. Available: http://kkmasterbatches.com/. [Accessed: 03-Feb-2019].
3 – Proposal 3: 2019. [Online]. Available: https://www.homedepot.ca/en/home/p.defender-1l-ready-to-use-garden-fungicide.1000107206.html. [Accessed: 04- Feb- 2019].
4 – Proposal 1: “Evolution Series,” Dream Pond. [Online]. Available: https://www.dreampond.com/koi-pond-water-garden/water-pumps/evolution-series.html. [Accessed: 04-Feb-2019].
4 – Proposal 2: Web-Know-How, “Water Delivery selection guide,” ROVER & ROVER Battery Single Point Watering System. [Online]. Available: http://www.aquapro.net/tank_info.html. [Accessed: 04-Feb-2019].
4 – Proposal 3: “Canilla PVC para manguera manija mariposa Ø ½ DUKE- www.deplano.com.ar,” DePlano. [Online]. Available: https://www.deplano.com.ar/canillas-pvc/2270-canilla-esf-pvc-mariposa-o-1-2-duke-60.html. [Accessed: 04-Feb-2019].
5 – Proposal 1: Buyers Products | Brands. [Online]. Available: https://www.surpluscenter.com/Electrical/AC-Motors/. [Accessed: 04-Feb-2019].
5 – Proposal 2: “NEMA 23 Stepper Motor | 3A , 30kg-cm,” DIYElectronics. [Online]. Available: https://www.diyelectronics.co.za/store/stepper-motors/478-nema-23-57bygh115-003.html. [Accessed: 04-Feb-2019].
5 – Proposal 3: "Hitec HS-645MG Servo Motor", RobotShop, 2019. [Online]. Available: https://www.robotshop.com/uk/hitec-hs-645mg-servo-motor.html. [Accessed: 04- Feb- 2019].
6 – Proposal 1: “Coffee Plants of the World,” Specialty Coffee Association. [Online]. Available: https://sca.coffee/research/coffee-plants-of-the-world/. [Accessed: 04-Feb-2019].
6 – Proposal 2: “100 litre Woven Planter Bags,” Nursery and Garden Supplies Australia. [Online]. Available: https://nurseryandgardensupplies.com.au/product/woven-planter-bags-100-ltr/. [Accessed: 04-Feb-2019].
6 – Proposal 3: “How to Grow Plants in Raised Beds,” Thompson & Morgan. [Online]. Available: https://www.thompson-morgan.com/how-to-grow-plants-in-raised-beds. [Accessed: 04-Feb-2019].
7 – Proposal 1: 2019. [Online]. Available: https://www.homedepot.ca/en/home/p.defender-1l-ready-to-use-garden-fungicide.1000107206.html. [Accessed: 04- Feb- 2019].
7 – Proposal 2: “Kingfisher Garden Pressure Sprayer 5L,” JTF. [Online]. Available: https://www.jtf.com/sprayer-pressure-5-l.html. [Accessed: 04-Feb-2019].
8 – Proposal 1: Hydroponic Gardening & More with Brent, “PVC Watering System,” YouTube, 31-Dec-2015. [Online]. Available: https://www.youtube.com/watch?v=AVWP7gjjka0. [Accessed: 04-Feb-2019].
8 – Proposal 2: M. Caffeine, “How To: Build A DIY Rain Machine For Under $20!,” YouTube, 18-Apr-2014. [Online]. Available: https://www.youtube.com/watch?v=XnHRB_P4F70. [Accessed: 04-Feb-2019].
8 – Proposal 3: Dl.sciencesocieties.org, 2019. [Online]. Available: https://dl.sciencesocieties.org/images/publications/jeq/35/6/2229fig2.jpeg. [Accessed: 04- Feb- 2019].
9 – Proposal 1: “Travesses de fusta,” Fusta de serra. [Online]. Available: https://www.fitorforestal.com/ca/travesses-de-fusta.html. [Accessed: 04-Feb-2019].
9 – Proposal 2: “MRC MERCANTILE,” MRC Mercantile Acrylic Plastics Cebu. [Online]. Available: http://acrylicsheetcebu.com.ph/. [Accessed: 04-Feb-2019].
9 – Proposal 3: “He Made This Ugly Cinderblock Wall Beautiful With MORE Cinderblocks! - Page 2 of 2,” Pinterest. [Online]. Available: https://co.pinterest.com/pin/163818505173203549/. [Accessed: 04-Feb-2019].

9 – Proposal 4: "Chopping Boards & Racks", Busy Chef, 2019. [Online]. Available: https://www.busychef.co.uk/collections/chopping-boards-racks/products/collection_-tablecraft-cutting-board. [Accessed: 04- Feb- 2019].
10 – Proposal 1: "Field Inoculation and Classification of Maize Ear Rot Caused by Fusarium verticillioides ," Bradford Protein Assay - BIO-PROTOCOL. [Online]. Available: https://bio-protocol.org/e3099. [Accessed: 04-Feb-2019].
10 – Proposal 2: Inspiring.vector.gmail.com, "diente de león blanco," Depositphotos. [Online]. Available: https://mx.depositphotos.com/111413150/stock-illustration-dandelion-on-white.html. [Accessed: 04-Feb-2019].
10 – Proposal 3: "Cómo limpiar las plantas," MiaRevista.es. [Online]. Available: https://www.miarevista.es/hogar/articulo/como-limpiar-las-plantas-481444999448. [Accessed: 04-Feb-2019].
10 – Proposal 4: "5 tuti tipp, hogy tisztán tartsd a szobanövényeidet," Gardenista, 03-Mar-2018. [Online]. Available: https://gardenista.hu/2018/03/04/5-tut-tipp-hogy-tisztan-tartsd-a-szobanovenyeidet/. [Accessed: 04-Feb-2019].
12 – Proposal 1: "What is the difference between sprinkler irrigation and furrow irrigation?", Quora, 2019. [Online]. Available: https://www.quora.com/What-is-the-difference-between-sprinkler-irrigation-and-furrow-irrigation. [Accessed: 04- Feb- 2019].
12 – Proposal 2: 2019. [Online]. Available: https://www.homedepot.ca/en/home/p.defender-1l-ready-to-use-garden-fungicide.1000107206.html. [Accessed: 04- Feb- 2019].
12 – Proposal 3: "Kingfisher Garden Pressure Sprayer 5L," JTF. [Online]. Available: https://www.jtf.com/sprayer-pressure-5-l.html. [Accessed: 04-Feb-2019].
12 – Proposal 4: Daisy Creek Farms with Jag Singh, "Drill Holes in 1/2' PVC Pipe and This Happens," YouTube, 08-May-2018. [Online]. Available: https://www.youtube.com/watch?v=hazWfD45wNM. [Accessed: 04-Feb-2019].
13 – Proposal 1: "Wind Speed and Direction Sensor | Dust Sentry & AQM 65," Aeroqual. [Online]. Available: https://www.aeroqual.com/product/gill-wind-sensor. [Accessed: 04-Feb-2019].
13 – Proposal 2: "Wind Speed/Direction, Modbus, 10 m Cable - IC-WSD-1-002," Instrument Choice. [Online]. Available: https://www.instrumentchoice.com.au/instrument-choice/weather-stations/proffesional-weather-stations/dyacon-weather-station-accessories-and-sensors/wind-speed-direction-modbus-10-m-cable-ic-wsd-1-002. [Accessed: 04-Feb-2019].
13 – Proposal 3: T. Twmffat and Instructables, "Arduino GPRS Weather Station - Part 2: Upgraded Wind Sensors and Improved Energy Efficiency," Instructables.com, 20-Jan-2019. [Online]. Available: https://www.instructables.com/id/Arduino-GPRS-Weather-Station-Part-2-Upgraded-Wind-/. [Accessed: 04-Feb-2019].
14 – Proposal 1: "water flow meter liquid flowmeter rotameter meter flow 94mm-in Flow Meters from Tools on Aliexpress.com | Alibaba Group," aliexpress.com. [Online]. Available: https://www.aliexpress.com/item/water-flow-meter-liquid-flowmeter-rotameter-meter-flow-94mm/32373639290.html. [Accessed: 04-Feb-2019].
14 – Proposal 2: "Pluviometer," Weer en klimaat. [Online]. Available: https://weerkenklimaat.weebly.com/pluviometer.html. [Accessed: 04-Feb-2019].
14 – Proposal 3: "Electro Magnetic Water Meter," IndiaMART.com. [Online]. Available: https://www.indiamart.com/proddetail/electro-magnetic-water-meter-17008786755.html. [Accessed: 04-Feb-2019].
15 – Proposal 1: "NEMA 23 Stepper Motor | 3A , 30kg-cm," DIYElectronics. [Online]. Available: https://www.diyelectronics.co.za/store/stepper-motors/478-nema-23-57bygh115-003.html. [Accessed: 04-Feb-2019].
15 – Proposal 2: "Hitec HS-645MG Servo Motor", RobotShop, 2019. [Online]. Available: https://www.robotshop.com/uk/hitec-hs-645mg-servo-motor.html. [Accessed: 04- Feb- 2019].
15 – Proposal 3: Micrologia, "con una sola mano tirando la cuerda sobre fondo blanco," Depositphotos. [Online]. Available: https://mx.depositphotos.com/27740207/stock-photo-single-hand-pulling-rope-on.html. [Accessed: 04-Feb-2019].
16 – Proposal 1: Ltd, "Dişli Zincir Dişli Zincir / Urunlerimiz / Zincir Kizaklari," Dişli Zincir / Türkiye. [Online]. Available: http://www.dislizincir.com.tr/Urunlerimiz/Zincir-Kizaklari. [Accessed: 04-Feb-2019].
16 – Proposal 2: D. Carpenter, "Curved Linear Rail," Ballscrews | 16mm & Below For Industrial Applications. [Online]. Available: https://www.tpa-us.com/curved-linear-rail.html. [Accessed: 04-Feb-2019].
16 – Proposal 3: "Konmison Sfu1605 Linear Guide Rail 400mm Linear Motion Rail Cnc Linear Rail Shaft Guide with Stepper Motor Nema 23 Support," Dorling Kindersley Science Encyclopedia: Amazon.co.uk: Dorling Kindersley Publishing: 9780751356410: Books. [Online]. Available: https://www.amazon.co.uk/Konmison-Sfu1605-Linear-Stepper-Support/dp/B016MC2W3C. [Accessed: 04-Feb-2019].
16 – Proposal 4: J. Petersen and L. Myers, "Wooden Train Freight Cars -- The Play Trains! Ultimate Wooden Train Guide," Play Trains!, 09-Dec-2017. [Online]. Available: https://play-trains.com/wooden-train-freight-cars-with-removable-freight/. [Accessed: 04-Feb-2019].
17 – Proposal 1: "Online Home Store for Furniture, Decor, Outdoors & More," Wayfair. [Online]. Available: https://www.wayfair.com/bed-bath/pdx/ben-and-jonah-clean-home-peva-single-shower-curtain-liner-banj1011.html. [Accessed: 04-Feb-2019].
17 – Proposal 2: Pam, Kristin, Toni, Hannah, Claire, C. Drake, and Denise, "DIY No-Sew Drop Cloth Curtains a CHEAP DIY Curtain Rod," The Crazy Craft Lady, 11-Jan-2019. [Online]. Available: https://thecrazycraftlady.com/no-sew-drop-cloth-curtains/. [Accessed: 04-Feb-2019].
17 – Proposal 3: "project," Variegateinteriors FALSE CEILING AND WINDOW BLINDS. [Online]. Available: http://www.variegateinteriors.com/false-ceiling-and-window-blinds. [Accessed: 04-Feb-2019].

19 – Proposal 1: D. Anthony, “Top 5: Best Drones for Real Estate Photography - MyDroneChoice,” My Drone Choice, 11-Jun-2017. [Online]. Available: https://www.mydronechoice.com/best-drones-real-estate-photography/. [Accessed: 04-Feb-2019].
19 – Proposal 2: J. Erickson, “Parasitic fungi and the battle against coffee rust disease,” University of Michigan News, 13-Nov-2015. [Online]. Available: https://news.umich.edu/parasitic-fungi-and-the-battle-against-coffee-rust-disease/. [Accessed: 04-Feb-2019].
20 – Proposal 1: “How To Install pH Sensors,” www.wateronline.com. [Online]. Available: https://www.wateronline.com/doc/how-to-install-ph-sensors-0001. [Accessed: 04-Feb-2019].
20 – Proposal 2: Turf-Tec Soil Compaction Tester / Dial Penetrometer. [Online]. Available: http://www.turf-tec.com/PHDS1lit.html. [Accessed: 04-Feb-2019].
20 – Proposal 3: “Measure Your Gut Health With a Stool pH Test,” Wellness Expert Specializing in Digestion & Joint Health. [Online]. Available: https://www.drdavidwilliams.com/measure-gut-health-with-stool-ph-test. [Accessed: 04-Feb-2019].
21 – Proposal 1: “Terms & Conditions,” Skye Instruments. [Online]. Available: http://www.skyeinstruments.com/news-events/lux-sensors/. [Accessed: 04-Feb-2019].
21 – Proposal 2: Zen Cart™ Team, “TSL2561 digital luminosity / lux / light sensor,” SK Pang Electronics Ltd. [Online]. Available: http://skpang.co.uk/catalog/tsl2561-digital-luminosity-lux-light-sensor-p-1131.html. [Accessed: 04-Feb-2019].
21 – Proposal 3: "Diode House +918030007370", Diode House +918030007370, 2019. [Online]. Available: http://diodehouse.in/ldr-light-dependent-resistor-sensor-ldr/p104. [Accessed: 04- Feb- 2019].
22 – Proposal 1: “DFROBOT Gravity: Analog Capacitive Soil Moisture Sensor- Corrosion Resistant,” Amazon. [Online]. Available: https://www.amazon.com/DFROBOT-Gravity-Capacitive-Corrosion-Resistant/dp/B01GHY0N4K. [Accessed: 04-Feb-2019].
22 – Proposal 2: R. Team, “DHT11 Temperature - Humidity Sensor Module : rhydoLABZ INDIA,” rhydoLABZ.com. [Online]. Available: https://www.rhydolabz.com/sensors-weather-sensors-c-137_147/dht11-temperature-humidity-sensor-module-p-2044.html. [Accessed: 04-Feb-2019].
22 – Proposal 3: “NEW PRODUCT – Soil Temperature/Moisture Sensor – SHT10,” Adafruit Industries - Makers, hackers, artists, designers and engineers!, 14-Mar-2013. [Online]. Available: https://blog.adafruit.com/2013/03/14/new-product-soil-temperaturemoisture-sensor-sht10/. [Accessed: 04-Feb-2019].
22 – Proposal 4: Soil Sensors. [Online]. Available: http://www.eagle-tek.com/en/category/products/f. [Accessed: 04-Feb-2019].
R. Team, “DHT11 Temperature - Humidity Sensor Module : rhydoLABZ INDIA,” rhydoLABZ.com. [Online]. Available: https://www.rhydolabz.com/sensors-weather-sensors-c-137_147/dht11-temperature-humidity-sensor-module-p-2044.html. [Accessed: 04-Feb-2019].
23 – Proposal 1: J. Shama, J. Shama, Forum News Service, and Forum News Service, “Tracking soil temperature an important step before planting,...,” Agweek, 21-Nov-2017. [Online]. Available: http://www.agweek.com/news/4254715-tracking-soil-temperature-important-step-planting-experts-say. [Accessed: 04-Feb-2019].
23 – Proposal 2: violetsupply.com. [Online]. Available: https://www.violetsupply.com/product/thermometer-humidity-gauge/. [Accessed: 04-Feb-2019].
23 – Proposal 3: R. Team, “DHT11 Temperature - Humidity Sensor Module : rhydoLABZ INDIA,” rhydoLABZ.com. [Online]. Available: https://www.rhydolabz.com/sensors-weather-sensors-c-137_147/dht11-temperature-humidity-sensor-module-p-2044.html. [Accessed: 04-Feb-2019].
23 – Proposal 4: Soil Sensors. [Online]. Available: http://www.eagle-tek.com/en/category/products/f. [Accessed: 04-Feb-2019].
R. Team, “DHT11 Temperature - Humidity Sensor Module : rhydoLABZ INDIA,” rhydoLABZ.com. [Online]. Available: https://www.rhydolabz.com/sensors-weather-sensors-c-137_147/dht11-temperature-humidity-sensor-module-p-2044.html. [Accessed: 04-Feb-2019].
24 – Proposal 1: “Gravity: Analog Electrical Conductivity Sensor / Meter(K=10),” DFRobot. [Online]. Available: https://www.dfrobot.com/product-1797.html. [Accessed: 04-Feb-2019].
24 – Proposal 2: Soil Sensors. [Online]. Available: http://www.eagle-tek.com/en/category/products/f. [Accessed: 04-Feb-2019].
25 – Proposal 3: T. Online, “The new 4GEE Capture Cam means you won't miss a thing,” T3, 01-Mar-2016. [Online]. Available: https://www.t3.com/news/the-new-4gee-capture-cam-means-you-wont-miss-a-thing. [Accessed: 04-Feb-2019].
26 – Proposal 4: “Steel metal ellipse frame border vector image on VectorStock,” VectorStock. [Online]. Available: https://www.vectorstock.com/royalty-free-vector/steel-metal-ellipse-frame-border-vector-3509355. [Accessed: 04-Feb-2019].
28 – Proposal 1: Compugold. [Online]. Available: http://www.plaza-tecnologia.com/mxProductDetails.php?id_product=105688. [Accessed: 04-Feb-2019].
28 – Proposal 2: “Kingston Digital 16GB microSDHC Class 10 UHS-I 45MB/s Read Card with SD Adapter (SDC10G2/16GB),” Amazon. [Online]. Available: https://www.amazon.com/Kingston-Digital-16GB-microSDHC-SDC10G2/dp/B0162YQEIE. [Accessed: 04-Feb-2019].
29 – Proposal 1: “Wireless Access Point PNG icons,” Free PNG and Icons Downloads. [Online]. Available: https://www.iconspng.com/image/9945/wireless-access-point. [Accessed: 04-Feb-2019].
29 – Proposal 2: ADXL345 - ADXL345 triple axis, digital accelerometer libr... | Mbed. [Online]. Available: https://os.mbed.com/cookbook/Xbee-Pro. [Accessed: 04-Feb-2019].
29 – Proposal 3: “Bluetooth Logo,” 1000 Logos The Famous Brands and Company Logos in the World. [Online]. Available: http://1000logos.net/bluetooth-logo/. [Accessed: 04-Feb-2019].

30 – Proposal 1: “How to Deal Wtih Missing Values in SAS,” SASCRUNCH TRAINING. [Online]. Available: https://www.sascrunch.com/dealing-with-missing-values.html. [Accessed: 04-Feb-2019].
30 – Proposal 2: “PUMAdb : Normalization Help,” Princeton University. [Online]. Available: https://puma.princeton.edu/help/results_normalization.shtml. [Accessed: 04-Feb-2019].
30 – Proposal 3: “Delete Blank Rows in Excel, Remove Blank Cells in Excel,” Chandoo.org - Learn Excel, Power BI & Charting Online, 25-Jan-2010. [Online]. Available: https://chandoo.org/wp/delete-blank-rows-excel/. [Accessed: 04-Feb-2019].
31 – Proposal 1: “Network data server 3d cloud computing concept realistic vector illustration,” City Of Forks City Limit Sign - FORKS / WASHINGTON. APRIL 13, 2017 Stock Footage Video 28374238 | Shutterstock, 04-Dec-2014. [Online]. Available: https://www.shutterstock.com/image-vector/network-data-server-3d-cloud-computing-235210087. [Accessed: 04-Feb-2019].
31 – Proposal 2: "Production line quality control management software - SCS Concept", SCS Concept, 2019. [Online]. Available: http://www.scsconcept.com/production-line-quality-control-management-software/. [Accessed: 04- Feb- 2019].
32 – Proposal 1: 2019. [Online]. Available: https://am207.github.io/2017/wiki/validation.html. [Accessed: 04- Feb- 2019].
33 – Proposal 1: CS231n Convolutional Neural Networks for Visual Recognition. [Online]. Available: http://cs231n.github.io/neural-networks-1/. [Accessed: 04-Feb-2019].
33 – Proposal 2: Mindmajix, “Learn How Support Vector Machine Algorithm Works With Examples,” Mindmajix, 11-Nov-2017. [Online]. Available: https://mindmajix.com/support-vector-machine-algorithm. [Accessed: 04-Feb-2019].
33 – Proposal 3: “How Random Forests Can Keep You From Decision Tree,” Sefik Ilkin Serengil, 12-Mar-2018. [Online]. Available: https://sefiks.com/2017/11/19/how-random-forests-can-keep-you-from-decision-tree/. [Accessed: 04-Feb-2019].
33 – Proposal 4: Kassambara, “CLARA - Clustering Large Applications,” Correlation matrix : A quick start guide to analyze, format and visualize a correlation matrix using R software - Easy Guides - Wiki - STHDA, 09-Apr-2017. [Online]. Available: http://www.sthda.com/english/articles/tag/pam-clustering/. [Accessed: 04-Feb-2019].
34 – Proposal 1: “Hinge loss,” Wikipedia, 15-Jan-2019. [Online]. Available: https://en.wikipedia.org/wiki/Hinge_loss. [Accessed: 04-Feb-2019].
34 – Proposal 2: S. Singla and S. Singla, “Experiments with a New Loss Term Added to the Standard Cross entropy,” medium.com, 09-Sep-2017. [Online]. Available: https://medium.com/mlreview/experiments-with-a-new-loss-term-added-to-the-standard-cross-entropy-85b080c42446. [Accessed: 04-Feb-2019].
34 – Proposal 3: MosheWorld, “MosheWorld/Machine-Learning,” GitHub. [Online]. Available: https://github.com/MosheWorld/Machine-Learning. [Accessed: 04-Feb-2019].
35 – Proposal 1: Sinteza Agro - WHY NOT?[Online]. Available: https://whynot.com.ua/en/portfolio/branding/sinteza_agro/. [Accessed: 04-Feb-2019].
35 – Proposal 2: “How to Setup Email Notification,” Geotab Blog. [Online]. Available: https://www.geotab.com/video/setup-email-notification/. [Accessed: 04-Feb-2019].
35 – Proposal 3: “How mobile apps are helping agriculture in achieving sustainable development?,” SourceTrace Systems, 26-Nov-2018. [Online]. Available: http://www.sourcetrace.com/mobile-apps-for-agriculture/. [Accessed: 04-Feb-2019].
35 – Proposal 4: “Developer Portal | Developer Portal : ThingWorx,” Brand. [Online]. Available: https://developer.thingworx.com/. [Accessed: 04-Feb-2019].
